# Peri-Implant Fractures Distal to an Antegrade Femoral Nail: A Case Series

**DOI:** 10.5704/MOJ.1603.012

**Published:** 2016-03

**Authors:** T Jegathesan, BK Ernest-Kwek

**Affiliations:** Department of Orthopaedics, Tan Tock Seng Hospital, Singapore

**Keywords:** Peri-implant fracture, femur intramedullary nail, polyaxiallocking plate

## Abstract

Peri-implant fractures distal to an antegrade femoral nail are uncommon injuries, with no current consensus on the best treatment modality. We are presenting three cases of periimplant fractures distal to an antegrade femoral nail. All patients sustained an initial traumatic injury, which was managed with an antegrade femoral nail fixation. They subsequently suffer a second injury which causes periimplant fracture. Our first two patients were managed with removing the intramedullary nail followed by Less Invasive Stabilization System (LISS) plate fixation. In our third case, the intramedullary nail was left in-situ, and the fracture was fixed with a polyaxial locking plate We discuss their injury pattern, investigations and surgical management. Polyaxial locking plates show great promise in this setting as they allow fixation of the fracture whilst maintaining the existing nail to protect the entire femur from further injury.

## Introduction

Antegrade intramedullary nailing of the femur is an effective treatment method for diaphyseal fractures.^1^ However, peri-implant fractures occurring distal to an antegrade femoral nail are uncommon, and present a daunting challenge for an orthopaedic surgeon. Distal femoral fractures can arise through two principal injury mechanisms. These are either high velocity trauma, such as road traffic accidents, which may involve comminution of the condyles and metaphysis, or low energy trauma in elderly patients with a background of osteoporosis.^2^

We report three cases of peri-implant fractures distal to an antegrade femoral nail. The patients highlighted in this case series all sustained an initial traumatic injury, which was similarly managed with antegrade femoral nail. In each of the three cases, they would later sustain a second subsequent traumatic injury. We discuss their injury pattern, investigations and surgical management.

## Case Report One

A 76 year-old man with no prior significant surgical history sustained a left proximal femur fracture after falling from a bicycle. Radiographs showed a closed, left femur subtrochanteric fracture (AO 32-A3). He underwent a long Gamma nail (Stryker, Japan) insertion with two distal interlocking bolts. His fracture went on to uncomplicated union and the patient was discharged from follow-up after attaining full functional recovery.

He was hospitalized again four years later after slipping on a wet floor, twisting his left leg in the process. Radiographs showed an oblique supracondylar fracture (AO 33-A1) at the level of the distal most interlocking bolt. The implants had no evidence of loosening. (Fig 1a(i,ii)).

**Fig. 1a:**
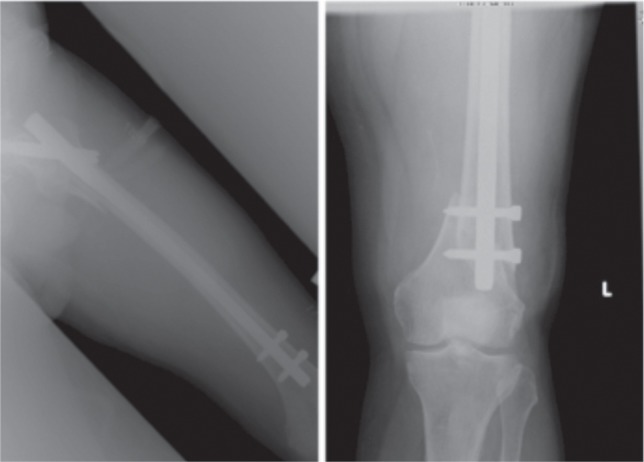
(i) Radiograph left femur after Gamma nail insertion. (ii) Radiograph left distal femur after 2nd admission.

Intraoperatively, the left intramedullary nail was removed first. Following which, the Less Invasive Stabilization System (LISS, Synthes, Paoli, PA) plate was inserted in a submuscular fashion to address the fracture distally.(Fig 1b) The patient was initiated on partial weight bearing, and progressed to full weight bearing after one month. He was last seen in the clinic a year after the second admission to hospital for the left distal femur supracondylar fracture, with radiographs showing union. He was then ambulating with a walking stick.

**Fig. 1b:**
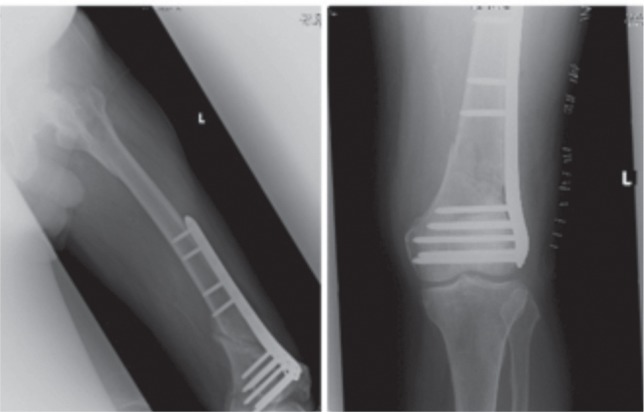
Radiographs after LISS plate insertion.

## Case Report Two

A 30 year-old man was admitted after a motor vehicle accident, sustaining a right mid femoral shaft fracture. He was operated upon and a piriformis entry standard locking intramedullary nail was inserted with two distal locking screws.(Fig 2a(i)) During outpatient follow up, complaints of right hip pain near insertion site eventually led to the removal of the proximal locking screw four years later. He was discharged from follow up after complete healing of the fracture.

The patient subsequently sustained multiple fractures. The patient involve in another accident and sustained polyfractures. The injury were distal femur comminuted fracture extending intra-articularly (AO 33-C1), with open proximal shaft fracture of the tibia and fibula. (Fig 2a(ii) Other fractures he sustained were a right femoral neck fracture, an open book pelvic fracture, a right clavicle fracture, left tibia shaft fracture and numerous metacarpal and metatarsal fractures of his left hand and foot respectively.

**Fig. 2a:**
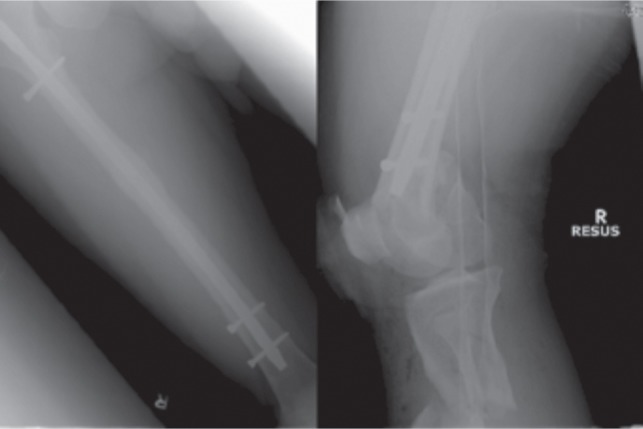
(i) Radiograph after intramedullary nail insertion. (ii) Radiograph right distal femur after polytrauma.

The neck of femur fracture was treated via cancellous screw fixation and the rest of his bilateral lower limb fractures were initially stabilized via external fixation and then definitively operated on four days after. After removal of the previous intramedullary nail, the Less Invasive Stabilization System (LISS, Synthes, Paoli, PA) plate was inserted to manage the distal femur comminuted fracture. Bilateral tibial fractures were also fixed in the same sitting. (Fig 2b)

**Fig. 2b:**
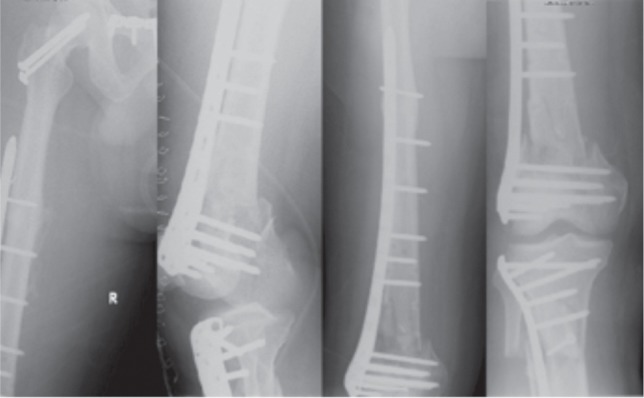
Radiographs post definitive fixation following polytrauma.

He eventually received autogenous bone grafting for non-union of his right distal femur and right tibia fracture. Latest radiographs at three years post-surgery showed callus formation, with the patient being able to ambulate with walking aid. The patient was subsequently lost to follow up.

## Case Report Three

A 62-year old male motorcyclist with no significant past medical history, sustained a right femur shaft fracture following a motor vehicle accident. Radiographs (Fig 3a(i)) revealed a transverse femur shaft fracture (AO 32-A3), which was subsequently reduced and fixed with a piriformis start standard locking intramedullary nail with two proximal and two distal interlocking screws. (Fig 3a(ii) Despite developing non-union of the right femur fracture, this patient was able to walk with tolerable pain and declined further surgical treatment.

**Fig. 3a:**
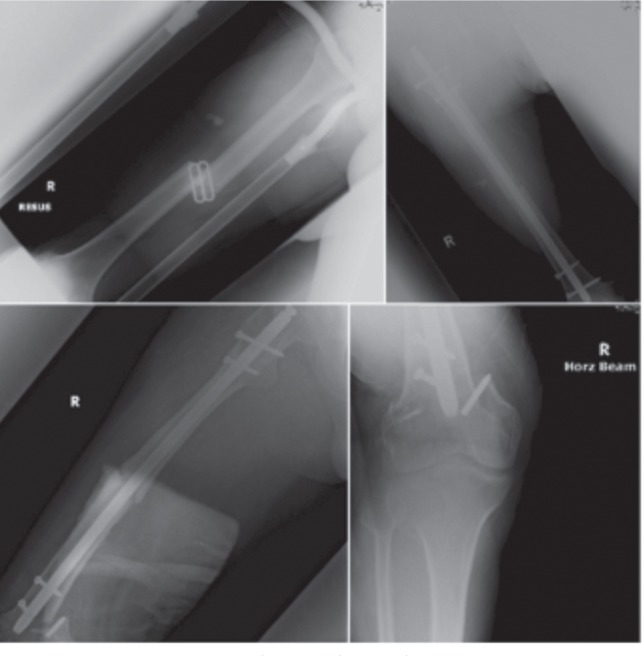
(i) Radiograph of initial femur fracture (ii) Radiograph post intramedullary nail insertion

However two years later, he was involved in another motor vehicle accident after his motorcycle collided with a car. Radiographs showed a displaced fracture of the right distal femur with intraarticular extension (AO 33-B2). There was no peri-implant loosening but there was breaking of the distal interlocking screw with the broken tip in bone. (Fig 3a(iii,iv) A CT scan showed a non-united fracture of the mid femoral shaft and a comminuted fracture of the femoral condyles.

He underwent open reduction and internal fixation of his right distal femur and tibial plateau two weeks after the accident to allow for the initial swelling to subside. The articular fragment was reduced and fixed with multiple 3mm headless screws. A polyaxial locking plate (Zimmer, Warsaw, Indiana) was tunnelled submuscularly and was secured with two locking screws proximally bypassing the femur nail. Variable angle screws were also inserted distally to bypass the nail. The incarcerated remnant of the interlocking bolt was left buried. The tibial plateau was fixed using an anatomical locking plate. Non-union of the previous right femur shaft was treated with autogenous bone grafts. He was started on full weight bearing after six weeks and last radiographs showed good union of all fracture sites. (Fig 3b)

**Fig. 3b:**
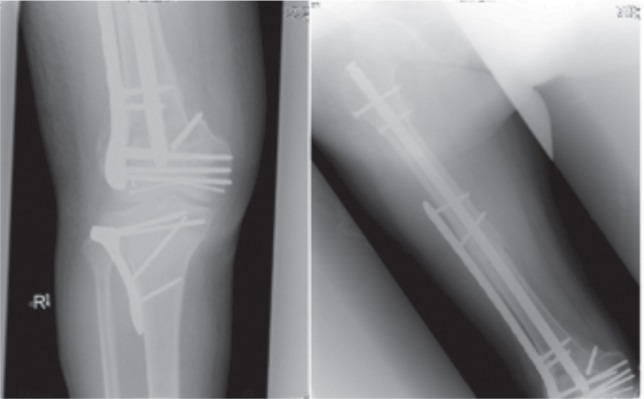
Radiographs post 2nd injury definitive fixation.

## Discussion

For the first patient who was of elderly age, it was noted that the initial intramedullary nail did not span the entire femur length, with approximately 3cm of the femur length distal to the nail being unprotected. Given the mechanism of injury for the second and third patients, the fracture for the first patient could be considered an osteoporotic fracture. It did not require a significant amount of trauma to result in a peri-implant fracture, which was extra-articular and in the metaphyseal region. Possible intrinsic risk factors for sustaining a peri-implant distal femur fracture mainly relate to the quality and quantity of bone present in the distal femur. These include advanced age, osteopenia/ osteoporosis, inflammatory arthropathy, chronic corticosteroid use, implant loosening and presence of infection. Such a case of a low energy peri-implant fracture raises evidence for prophylactic nailing of the femur in cases of osteoporosis.

The underlying mechanism of the later injuries in the second and third patients, following their respective antegrade nail insertion, involved accidents of immense velocity resulting in the peri-implant fractures. These two cases illustrate that especially in high energy trauma, antegrade nails do not always protect the entire femur from subsequent bony injury. Fractures distal to the nail, especially involving the femoral condyles, can still occur.

In our first two patients, management of the peri-implant fractures encompassed a similar method the intramedullary nail was removed prior to fixation via a LISS plate. This however, potentially leaves the proximal aspect of the femur unprotected. In contrast, in our third patient, the intramedullary nail was left *in-situ*, and a polyaxial locking plate was inserted for the peri-implant fracture. In our third patient where the initial fracture site showed non-union, the peri-implant fracture was managed without removing the intramedullary nail. The polyaxial distal femur plate combines conventional plating technique with polyaxial screw placement and angular stability. Intramedullary implants are bypassed by the screw and angular stability is achieved by fixing the head of the screw with an additional cap threaded into the plate. Such a technique has shown promising results regarding union and mal-union rates in periprosthetic fractures in elderly and osteoporotic patients. This thus provides a good alternative to the standard locking plate when faced with challenges of fixing fractures that have an intramedullary nail in situ.

In a study done by Ruchholtz *et al*^3^, 41 periprosthetic and peri-implant femur fractures underwent similar surgical fixation with an anatomical polyaxial locking plate system with no failure of fixation reported in the study patients. The authors recommended using a long plate to achieve sufficient number of screws on both sides of the fracture, and suggested an additional cerclage in order to achieve optimal stability when fewer than four screws could be set. Another recent study by Hoffman *et al*^4^ also evaluated interprosthetic femoral fractures with polyaxial locking plate surgical treatment with 89% union rate.

## Conclusion

Peri-implant fractures distal to an antegrade femoral nail are rare injuries, with no current consensus on the best treatment modality. Effective management of peri-implant fractures of the distal femur requires careful assessment of displacement, fracture location, stability of implants and adequacy of distal bone stock. However, polyaxial locking plates do show great promise in this setting as they allow fixation of the fracture whilst maintaining the existing nail to protect the entire femur from further injury.

## References

[1] Wolinsky P, Tejwani N, Richmond JH, Koval KJ, Egol K, Stephen DJ (2002). Controversies in Intramedullary Nailing of Femoral Shaft Fractures. Instr Course Lect.

[2] Link BC, Babst R (2012). Current concepts in Fractures of the Distal Femur. Acta Chir Orthop Traumatol Cech.

[3] Ruchholtz S, El-Zayat B, Kreslo D, Bucking B, Lewan U, Kruger A (2013). Less Invasive Polyaxial Locking Plate Fixation in Peri-prosthetic and Peri-implant Fractures of the Femur – A Prospective Study of 41 Patients. Injury.

[4] Hoffmann MF, Lotzien S, Schildhauer TA (2016). Clinical Outcome of Interprosthetic Femoral Fractures Treated with Polyaxial Locking Plates. Injury.

